# Sex and Age Don't Matter, but Breed Type Does—Factors Influencing Eye Wrinkle Expression in Horses

**DOI:** 10.3389/fvets.2019.00154

**Published:** 2019-05-29

**Authors:** Lisa Schanz, Konstanze Krueger, Sara Hintze

**Affiliations:** ^1^Division of Livestock Sciences, Department of Sustainable Agricultural Systems, University of Natural Resources and Life Sciences, Vienna, Austria; ^2^Department of Equine Economics, Nuertingen-Geislingen University of Applied Sciences, Nürtingen, Germany; ^3^Biology I, University of Regensburg, Regensburg, Germany

**Keywords:** horse, welfare, facial expression, eye, eye wrinkles, individual characteristics, breed type

## Abstract

Identifying valid indicators to assess animals' emotional states is a critical objective of animal welfare science. In horses, eye wrinkles above the eyeball have been shown to be affected by pain and other emotional states. From other species we know that individual characteristics, e.g., age in humans, affect facial wrinkles, but it has not yet been investigated whether eye wrinkle expression in horses is systematically affected by such characteristics. Therefore, the aim of this study was to assess how age, sex, breed type, body condition, and coat colour affect the expression and/or the assessment of eye wrinkles in horses. To this end, we adapted the eye wrinkle assessment scale from Hintze et al. (1) and assessed eye wrinkle expression in pictures taken from the left and the right eye of 181 horses in a presumably neutral situation, using five outcome measures: a qualitative first impression reflecting how worried the horse is perceived by humans, the extent to which the brow is raised, the number of wrinkles, their markedness and the angle between a line through both corners of the eye and the topmost wrinkle. All measures could be assessed highly reliable with respect to intra- and inter-observer agreement. Breed type affected the width of the angle [*F*_(2,114)_ = 8.20, *p* < 0.001], with thoroughbreds having the narrowest angle (*M* = 23.80, *SD* = 1.60), followed by warmbloods (*M* = 28.00, *SD* = 0.60), and coldbloods (*M* = 31.00, *SD* = 0.90). None of the other characteristics affected any of the outcome measures, and eye wrinkle expression did not differ between the left and the right eye area (all *p*-values > 0.05). In conclusion, horses' eye wrinkle expression and its assessment in neutral situations was not systematically affected by the investigated characteristics, except for “breed type”, which accounted for some variation in “angle”; how much eye wrinkle expression is affected by emotion or perhaps mood needs further investigation and validation.

## Introduction

Assessing emotional states in animals is a critical goal in animal welfare science, but it is generally agreed that the subjective experience of an emotion cannot be assessed directly [but see (2) for a different point of view]. Emotional states are multifaceted, including not only the subjective experience but also behavioural, physiological, and cognitive components, which can be assessed objectively and could therefore serve as indicators to infer animals' subjective experience [e.g., (3)]. Ideally, such indicators can be assessed non-invasively as well as reliably across various contexts, and do not require the animals to be trained. Spontaneous behaviour, including facial expressions, are promising examples of indicators to assess animals' emotional states.

Facial expressions in animals have mainly been studied using Facial Action Coding Systems (FACSs) and Grimace Scales (GSs). In FACSs, all possible facial muscle movements and resulting expressions are systematically catalogued as Action Units or Action Descriptors (4). Originally developed for humans, FACSs have now been adapted to different animal species, including primates [orangutans (5), macaques (6), chimpanzees (7, 8), gibbons (9)], dogs (10), cats (11), and horses (12). Besides their application in comparative psychology [e.g., (13)] and research on the evolution of emotional communication [e.g., (14)], FACSs have more recently also been used to associate facial expressions with emotional states (11).

Grimace Scales have been developed for the assessment of pain by comparing the facial expressions of animals in painful and pain free conditions and systematically identifying the changes. They exist for a range of species, including laboratory animals [e.g., mice (15); rats (16); rabbits (17)], farm animals [e.g., sheep (18), including lambs (19), piglets (20)] and horses (21). In horses, two scales, namely the Horse Grimace Scale (HGS, (21)) and the Equine Pain Face (EPF, (22)) have been published. These scales have been developed by comparing the facial expressions of horses before and after castration while undergoing different pain treatments (HGS) and horses exposed to two noxious stimuli expected to induce pain (EPF). Both studies identified, beyond several other Action Units, change above the horses' eyes as more prevalent when horses were in pain (HGS: “tension above the eye area”, EPF: “angled eye”). One aspect of this change above the eye is the appearance of eye wrinkles, often dubbed “worry wrinkles” in the equine community, thereby suggesting an association with negative emotions. To investigate whether eye wrinkle expression is affected by emotional states, Hintze et al. (1) developed a detailed scale for this expression caused by the contraction of the levator anguli oculi medialis and corrugator supercilii muscles and the resultant raised inner brow [identified as Action Unit 101 in EquiFACS; (12)]. This scale describes different characteristics of the wrinkles, including the number and markedness of wrinkles and the angle between a line through both corners of the eye and the topmost wrinkle. The scale was applied to pictures of sixteen horses, which were exposed to two presumably positive situations (grooming, food anticipation) and two presumably negative situations (waving of plastic bag, food competition) in a counterbalanced order. It was found that the angle decreased during grooming (muscle relaxation) and increased during food competition (muscle contraction) compared to control phases but no other characteristics of the eye wrinkle expression were systematically affected by these situations.

The results of this first study on the association between eye wrinkle expression and horses' emotional states are promising with respect to the angle measure, but further validation is needed before eye wrinkles can be used as a potential indicator of emotional valence in horses. Furthermore, in some horses, eye wrinkles were already present in the neutral control phases and only the relative change of eye wrinkles between control and treatment phases was investigated and not the presence or absence of wrinkles. In case of a prolonged presence of eye wrinkles, it is unlikely that they are only influenced by the relatively quick contraction and dilation of the underlying facial muscles, but they may rather be facial landmarks. The contraction of the underlying facial muscles might interact with these wrinkles by leading to a more pronounced expression, for example, a deepening of already existing wrinkles (12). From humans and other animals we know that facial landmarks can be affected by individual characteristics. Facial wrinkles have, for example, been used to identify individual white rhinos (23) and to determine age in humans (24, 25). Moreover, the morphology of human faces differs between females and males, with male eyebrow ridges being more protruding than female ones (26). It has been suggested that the interaction between individual differences in facial landmarks and muscle movements, while often overlooked, does affect the assessment of facial expressions [e.g., (13, 27)]. In our study, we aimed to investigate whether eye wrinkle expression in horses is systematically affected by the horses' individual characteristics age, sex and breed type, since such characteristics might impact the assessment of emotional situations. Moreover, we were interested in studying whether body condition affects eye wrinkle expression, since there is anecdotal evidence that a pronounced hollow above horses' eyes is more prevalent in thin horses and may affect the expression of the wrinkles. In addition, factors that may not influence the expression itself, but the observer's perception and therefore the assessment of the expression, need to be considered. Human visual perception of, for example, depth, is influenced by brightness and colour (28), which may interfere with the assessment of facial expressions. Coat colour may be such a factor affecting the visibility of single wrinkles or their depth. For example, according to Dalla Costa et al. (21), it is easier to score lighter horses compared to darker horses for Action Units of the Horse Grimace Scale. Besides the described potentially influencing factors, it is important for the interpretation of the assessment whether eye wrinkle expression differs between the left and the right eye area of a horse. Humans show, for example, asymmetrical/unilateral facial expressions (29, 30). So far, there has been limited research on facial asymmetry in horses. One study showed that horses that were groomed in a gentle manner (assumed to elicit positive emotions) showed asymmetric ears less often than horses groomed in a standard procedure [assumed to elicit negative emotions; (31)].

If any of the aforementioned characteristics systematically affect eye wrinkle expression or its assessment, this needs to be considered when using eye wrinkles as a potential indicator of horses' emotional states. Consequently, we investigated how age, sex, breed type, body condition and coat colour affect the expression and/or assessment of eye wrinkles in horses, and whether the expression differs between the area above the left and right eye. To this end, we adapted the eye wrinkle assessment scale from Hintze et al. (1) to assess the individual characteristics on eye wrinkle expression in pictures of the left and right eye of 181 horses in a presumably neutral situation.

## Animals, Material and Methods

### Animals and Housing

This study included 181 horses (70 mares, 62 geldings, and 49 stallions) of varying age and breed (Supplementary Table 1). The horses' age ranged from 4 months to 28 years [mean (*M*) = 11.94, standard deviation (*SD*) = 6.48] and 44 different breeds and crossbreeds were included. Each horse was assigned to one of four “breed types” according to the breeds' stud books or official breed website when a stud book did not presently exist: coldblood (*n* = 36), warmblood (*n* = 104, including Quarter horses, Appaloosas, Paints, Fresians), thoroughbreds (*n* = 17, including Arabians, Trotters), ponies (*n* = 7), and one horse, which was of unknown breed. Breed crosses with breeds assigned to different breed types (e.g., Quarter horse × thoroughbred, draft × thoroughbred, Pinto × pony) were assigned to the breed type the horse phenotypically represented.

Horses were derived from seven farms across three countries: five farms in Germany (Farms 1–5), one farm in the United States of America (Farm 6), and one farm in Switzerland (Farm 7). Housing conditions varied between and within farms with horses kept either in standard single boxes, single paddock boxes or in groups. Horses in standard single boxes were kept on wood shavings, straw or a mixture of both with visual and in some cases physical contact to conspecifics. Horses living in a paddock box were also kept individually but with more space (box plus paddock) and physical contact to conspecifics was always possible. All horses kept in boxes were turned out either on paddocks or pastures, depending on the weather, in groups of at least two, except for stallions on Farm 7 and two older stallions on Farm 6, which were turned out individually. When kept in groups, horses were housed either in a pen system or on pasture, each with a shelter and a bedded lying area. Horses on all farms were fed hay and concentrates with the number of feedings per day varying between farms (hay: ranging from one feeding a day to hay *ad libitum*, concentrates: ranging from one to three feedings). On all farms, *ad libitum* access to water was provided by automatic drinkers, except for Farm 6, where water was supplied in buckets in the stables and big tanks on pastures. All horses were either exercised (riding, carriage driving), turned out on paddock or pasture, longed, or walked in a horse walker daily.

### Data Collection

#### Pictures Taken From the Eye Area

Data were collected on Farms 1–6 during spring and summer 2015, and on Farm 7 in summer 2014 and spring 2016. Horses were photographed in a presumably neutral situation in their habitual environment between 9 a.m. and 5 p.m. either in their box or on paddock/pasture. If any disturbance (visual or acoustic) occurred, photographing was stopped until the disturbance subsided. The photographer and/or the handler were familiar with the horse and both were instructed to interact as little as possible with the horse to keep the influence of handling to a minimum. The photographer stood at a 45° angle to each horse's head, while the handler was loosely holding the horse's halter. Several pictures of the horses' left and right eye areas were taken with a Canon G1X camera by one photographer (Farms 1–6) and with a Nikon D200 with telephoto lenses (Nikon AF Micro-Nikkor 60 mm f/2.8D and Nikon 80–200 mm f/2.8 AF-D) by two photographers (Farm 7). Pictures from the left and right hemiface were taken one after the other; which eye was photographed first was random but balanced across horses.

#### Body Condition Score (BCS)

After pictures from both eyes had been taken, the body condition of each horse was assessed by visual and tactile evaluation using the scale developed by Henneke et al. (32). With this scale, the presence or absence of adipose tissue and the visibility of bone structures is assessed on a nine-point scale (1–9, half points can be given). One person assessed the body condition of horses on all farms except for Farm 7, where the assessment was done by another person with the same scale. Agreement between the two assessors could not be evaluated due to large spatial and temporal distances of data collection. Sixteen horses on Farm 7 were not assessed. The BCS ranged from 2.5 to 8 (*M* = 5.3, *SD* = 0.9).

### Picture Processing

From all pictures we excluded blurry pictures and pictures in which the eye area was not fully visible from further assessment. Pictures were defined as blurry if wrinkles were not clearly detectable or the beginning and/or end of wrinkles was not visible (1). From the remaining pictures (*n* = 2,259), two or three pictures per horse and eye (left, right) were randomly selected for scoring (62 horses × 4 pictures and one horse with one eye × 2 pictures, data collection in summer 2014; 118 horses × 6 pictures, data collection in spring/summer 2015 and spring 2016) using the “sample” function in R (R Version 3.5.1, R Studio Version 1.1.453) and resulting in a total of 958 pictures. The selected pictures were cropped to only show the eye area needed for scoring and picture size was standardised using Microsoft Picture Manager (version 2018.18051.17710.0).

### Eye Wrinkle Assessment Scale

**Figure 1:**
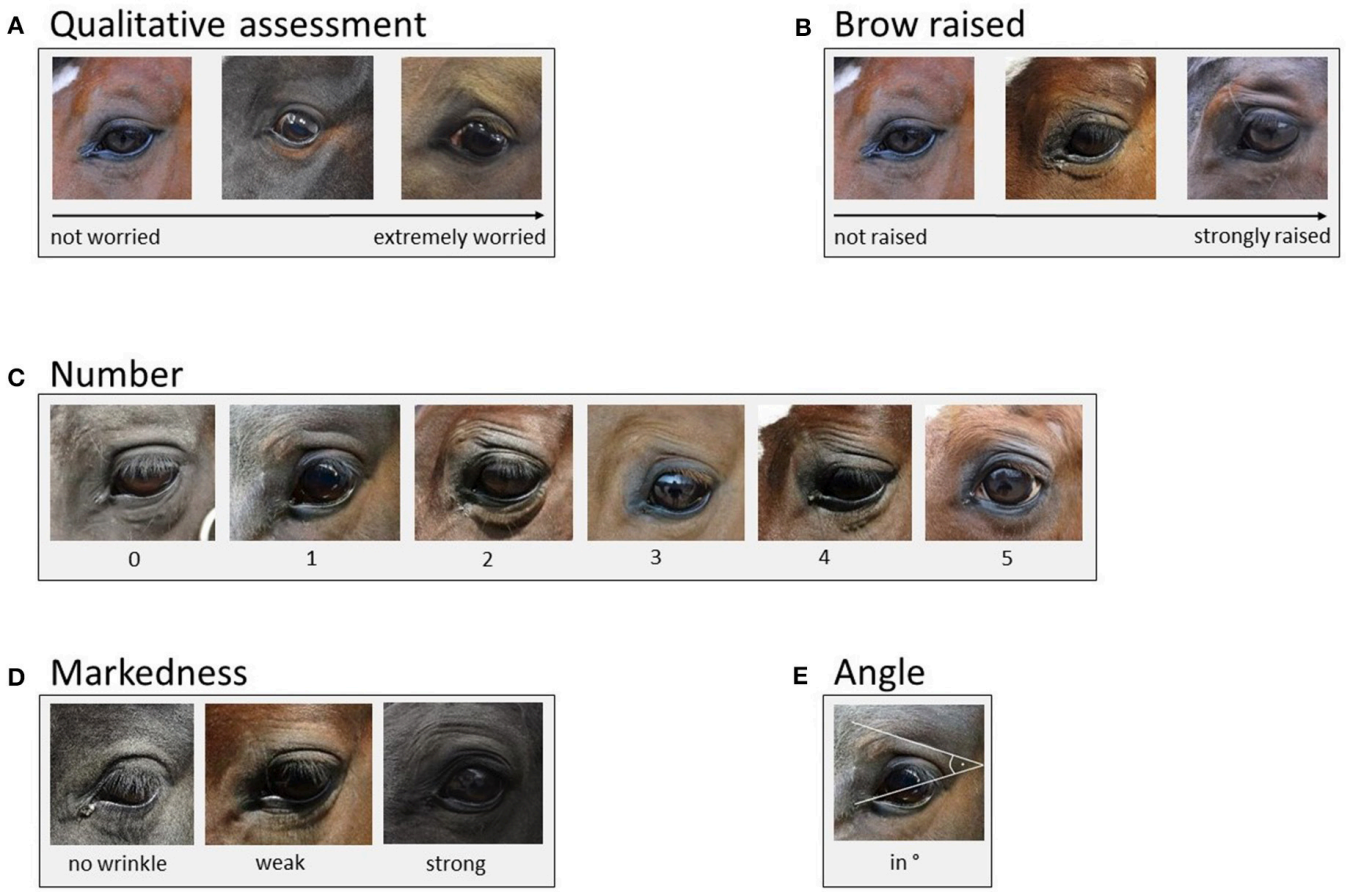
Eye wrinkle assessment scale [adapted from (1)]. **(A)** Qualitative assessment: The overall first subjective impression of the eye area with respect to how “worried” the horse actually looks as assessed on a Visual Analogue Scale ranging from “not worried” to “extremely worried.” **(B)** Brow raised: The amount the skin above the eye (brow region in humans) is raised, assessed on a Visual Analogue Scale ranging from “not raised” to “strongly raised.” **(C)** Number: Only wrinkles above the eye and those of a minimum length of one third of the eyeball's diameter are considered. A deep indent, often seen in thin horses, is not considered as a wrinkle (as it is not caused by the contraction of the muscles underlying the inner brow raiser). Moreover, wrinkles originating on the eyelid are not counted. **(D)** Markedness: The depth and width of the wrinkles is assessed. If the markedness differs between wrinkles, the most prominent wrinkle is assessed. “No wrinkle”: no wrinkle visible. “Weak”: wrinkles are flat and narrow lines. “Strong”: wrinkles are pronounced in depth and width. **(E)** Angle: The degree of the angle is measured at the intersection of the extension of a line drawn through the eyeball and the extension of the topmost wrinkle. The line through the eyeball extends from the medial to the lateral corner of the eyeball. If the medial corner is not clearly defined, the line goes through the middle of the tear duct.

In the present study, we used an adapted version of the eye wrinkle assessment scale developed by Hintze et al. (1) with five outcome measures: “qualitative assessment”, “brow raised”, “number”, “markedness”, and “angle” (for definitions and scoring details see Figure 1). “Number” (C), “markedness” (D), and “angle” (E) were defined as previously suggested by Hintze et al. (for a direct comparison of the two scales see Table 1). For the outcome measure “qualitative assessment” (A), we adapted the definition by Hintze et al. (1). The original definition focused on three particular aspects of the eye wrinkle expression, namely “the number […], markedness, and the angle” [1, p. 6], whereas in the present study we aimed to better capture the overall first subjective impression with respect to how “worried” the horse actually looks (“not worried” to “extremely worried”) without focusing on other aspects at this stage. Moreover, instead of using an ordinal scale with three distinct categories for “qualitative assessment”, we used Visual Analogue Scales (VAS) to possibly get more sensitive measures (33). VAS are an instrument to measure variables that range across a continuum of values (34). They are presented as a horizontal line with the end anchors labelled as the boundaries of this variable (e.g., “not worried” to “extremely worries”). For each variable (e.g., “worriedness”) a vertical mark through the line is placed at a position, which is deemed appropriate by the observer (34). We used the freely available programme AVAS (Adaptive Visual Analog Scales; 500 pixels, 132 mm, 0–100), which stores the positions of the mark on the line in an excel sheet (35). VAS were also used to assess the outcome measure “brow raised” (B), which replaced “eyelid shape” used in the scale described by Hintze et al. (1). Inter-observer agreement for “eyelid shape” was only moderate in the study by Hintze et al. (1). During discussions aiming to improve the definition and thereby inter-observer agreement, we realised that we could capture the amount the skin above the eye (the eyebrow region in humans) is pulled dorsally and obliquely in the direction of the medial frontal region [resembling Action Unit 1 in human FACS, (12)] more easily than the shape of the eyelid and consequently replaced “eyelid shape” with what we named “brow raised” (“not raised” to “strongly raised”). Moreover, we excluded the binary outcome measures “eye white” used by Hintze et al. (1) to assess the presence or absence of visible sclera, since our study focused on the effect of horses' characteristics on eye wrinkle expression in neutral situations and not on general changes in the eye area caused by different emotional situations.

**Table 1:** Comparison between the outcome measures used in the study by Hintze et al. (1) and in the present study.

**Hintze et al**. **(****1****)**		**Present study**		**Explanation for why (the definition of) the respective outcome measure was changed**
**Outcome measure**	**Assessment**	**Outcome measure**	**Assessment**	
Qualitative assessment	3 categories: “no wrinkles”, “medium”, “strong”	Qualitative assessment	VAS: ranging from “not worried” to “extremely worried”	Use of VAS because a continuous scale is potentially more sensitive than an ordinal scale (33).
Eyelid shape	3 categories: “round”, “weakly pulled”, “strongly pulled”	Brow raised	VAS: ranging from “not raised” to “strongly raised”	Only moderate inter-observer agreement for “eyelid shape” in Hintze et al. (1). “Brow raised” seems to better capture the visible change in the eye area. Use of VAS because it is potentially more sensitive than an ordinal scale and can improve inter-observer agreement (33).
Number	Continuous	Number	Continuous	Definition unchanged.
Markedness	3 categories: “no wrinkle”, “weak”, “prominent”	Markedness	3 categories: “no wrinkle”, “weak”, “prominent”	Definition unchanged.
Angle	Continuous in degrees	Angle	Continuous in degrees	Definition unchanged.
Eye white	Binomial category			Not used in the present study.

### Scoring

All 958 pictures were scored in random order by LS. The outcome measure “qualitative assessment” (assessed on a VAS) was scored first for all pictures to capture the first subjective impression of the eye wrinkles before assessing “brow raised” (also assessed on a VAS), followed by “number”, “markedness”, and “angle” [measured with CorelDraw, (36)]. LS was aware of the aim of the study, but was blind to all investigated effects with the exception of coat colour, for which we could not blind.

### Intra- and Inter-observer Agreement

To assess both intra- and inter-observer agreement, a sample of all pictures was re-scored by the same rater (LS, to assess intra-observer agreement) and by a second rater (SH, to assess inter-observer agreement). To assess intra-observer agreement, ten out of each subset of 50 pictures (in total *n* = 192 pictures) were randomly selected and scored a second time, at the earliest the day after the first scoring. To assess inter-observer agreement, a sample of 10% of all pictures (*n* = 96 pictures) was scored by SH after LS had finished scoring. Both raters were experienced in using the original and adapted eye wrinkle assessment scale.

### Coat Colour

The horses' “coat colour” was assessed by inspecting the area above the eye relevant for the eye wrinkle assessment in each picture. One of the following colours was assigned to each picture: black/dark bay, medium bay, light bay, grey/white, or undefinable (e.g., more than one colour present in the relevant area). Both raters (LS, SH) assessed all pictures together and at the same time, assigning a coat colour to each picture.

### Statistical Analysis

All analyses were performed with the statistical programming language R [R version 3.5.1, (37); RStudio version 1.1.453, (38)]. The data set can be found in Supplementary Table 2.

#### Intra- and Inter-observer Agreement

For continuous outcome measures (“qualitative assessment”, “brow raised”, “number”, “angle”), we calculated the Intraclass Correlation Coefficient [ICC, function: icc, package: irr, (39)] to assess agreement between first and second scoring of rater LS (intra-observer agreement) as well as between rater LS and SH (inter-observer agreement). For both intra- and inter observer agreement, we used a two-way mixed model with “single rater” as “type” and assessing absolute agreement (40). A *p*-value lower than 0.05 and an ICC-value above 0.75 were considered “excellent agreement” (41). Lower and upper bounds of the 95% confidence interval (CI) were assessed as a measure of deviation of the ICC. For the categorical outcome measure “markedness”, we calculated the association between two scorings with Cohen's Kappa [function: kappa2, package: irr, (39)], considering a *p*-value lower than 0.05 and a κ-value above 0.8 as “almost perfect agreement” (42).

#### Collinearity Between Explanatory Variables

In a statistical model, collinearity of explanatory variables can affect model interpretation and increase the standard errors of the coefficients (43). To explore the relationship between the different explanatory variables (“age”, “sex”, “Body Condition Score”, “breed type”, “coat colour”), each combination was tested for independence. For two continuous variables a Pearson's product-moment correlation coefficient was computed with 0.7 as the cut-off value for a “strong correlation” (44). When testing two categorical variables (e.g., “sex” and “breed type”), a Crammer's V test was performed (45). If V was above 0.7, only one of the two associated variables was included for further analyses. When testing one categorical and one continuous variable (e.g., “sex” and “age”), a Kruskal-Wallis test was run; if it reached statistical significance (*p* ≤ 0.05), a pairwise Wilcoxon rank sum test was performed as a *post-hoc* test to identify the levels of the categorical variable that differed from each other. If the results of the Wilcoxon rank sum test were statistically significant at all levels (*p* ≤ 0.05), only one of the associated variables was chosen for further analyses. This was the case for the association between “BCS” and “breed type”, as the mean “BCS” differed significantly across “breed types” (Kruskal-Wallis test: $\chi_{2}^{2}$ = 145.99, *p* < 0.001; pairwise Wilcoxon rank sum test: all three *p*-values < 0.01) with coldbloods having the highest BCS (*M* = 6.00, *SD* = 0.70), followed by warmbloods (*M* = 5.10, *SD* = 0.70), and thoroughbreds (*M* = 4.80, *SD* = 0.90) having the lowest BCS. Additionally, “BCS” and “sex” were associated (Kruskal-Wallis test: $\chi_{2}^{2}$ = 95.18, *p* < 0.001; pairwise Wilcoxon rank sum test: all three *p*-values < 0.01) with stallions (*M* = 5.90, *SD* = 0.90) having a greater “Body Condition Score” than both mares (*M* = 5.20, *SD* = 0.80) and geldings (*M* = 5.10, *SD* = 0.70). Since “BCS” had been assessed by two experimenters without testing for inter-observer agreement, we kept “breed type” and “sex” as the more reliable variables and excluded “BCS” from all further analyses.

#### Association Between Outcome Measures

All outcome measures (“qualitative assessment”, “brow raised”, “number”, “markedness”, “angle”) were tested for association. First a Kruskal-Wallis test, and if statistically significant a pairwise Wilcoxon rank sum test, was run to test for associations between “markedness” and all continuous measures. “Qualitative assessment” ($\chi_{2}^{2}$ = 301.92, *p* < 0.001), “brow raised” ($\chi_{2}^{2}$ = 333.06, *p* < 0.001) and “number” ($\chi_{2}^{2}$ = 881.66, *p* < 0.001) were associated with “markedness”; *p*-values < 0.01 for all pairwise Wilcoxon rank sum tests. Since “markedness” was a categorical measure on an ordinal scale, it was probably the least sensitive measure, and we therefore dropped it. All remaining outcome measures were continuous variables, and a Pearson's product-moment correlation coefficient was computed. If the correlation coefficient was > 0.7, indicating a “strong correlation” (44), only one outcome measure was selected for further analyses. A strong positive correlation between the outcome measures “qualitative assessment” and “brow raised” was found (*r* = 0.9, *p* < 0.001), and we selected “qualitative assessment” for all further analyses.

#### The Effect of the Different Explanatory Variables on the Outcome Measures

We employed a stepwise backwards selection procedure [functions: train, trainControl, packages: leaps, (46); caret, (47); MASS, (48)] based on the Akaike Information Criterion (AIC) to identify potentially relevant explanatory variables (“age”, “sex”, “breed type”, and “coat colour”). The stepwise backwards selection procedure indicates which variables are included in the best fitting model (49, 50). We decided to include all categorical explanatory variables in the final model from which at least one level was selected as potentially relevant. After selecting all potentially relevant explanatory variables, we analysed the effect of these variables on the continuous outcome measures (“qualitative assessment”, “number”, and “angle”) by running linear mixed-effects models [function: lme, package: nlme, (51)]. Explanatory variables selected as potentially relevant by the stepwise backwards selection procedure were included as fixed effects in the respective models, while random effects were included in all models as “eye” nested in “horse” nested in “farm” (see Table 2 for an overview of all effects). To verify model assumptions, the residuals for each executed model were visually checked for normal distribution and homogeneity of variance. No transformation of the data was necessary. *Post-hoc* tests were performed with the function lsmeans [package: lsmeans, (52)] using the “tukey method” to correct for multiple testing.

**Table 2:** Overview of all fixed and random effects, whether they were treated as continuous or as categorical variables, as well as their ranges [mean (*M*), standard deviation (*SD*)] for continuous variables and their levels for categorical variables.

**Effect**	**Fixed or random**	**Type of variable**	**Range (*M, SD*)/levels**
Age	Fixed (if selected)	Continuous	4 months−28 years (*M* = 11.90, *SD* = 6.50)
Sex	Fixed (if selected)	Categorical	3 levels (mare, gelding, stallion)
Breed type	Fixed (if selected)	Categorical	4 levels (coldblood, warmblood, thoroughbred, pony)
Coat colour	Fixed (if selected)	Categorical	4 levels (black/dark bay, medium bay, light bay, grey/white)
Farm	Random	Categorical	7 levels (Farms 1–7)
Horse	Random	Categorical	181 levels (horse 1–181)
Eye	Random	Categorical	2 levels (left, right)

#### Side Effects

To test for a possible difference between the right and the left eye, data were averaged per eye and horse by calculating the mean score of the respective pictures [function: aggregate, base R, (37)]. We then ran a linear mixed-effects model [function: lme, package: nlme, (51)] for all outcome measures with “eye” as fixed effect and “horse” nested in “farm” as random effects. Model assumptions were verified by visually checking residuals for normal distribution and homogeneity of variance. No transformation of the data was necessary.

## Results

### Sample Size and Data Structure

In total 958 pictures were assessed, including 118 horses with six pictures (three per eye), 62 horses with four pictures (two per eye) and one horse with only two pictures. Some pictures in the final sample could not be assessed reliably for all outcome measures due to low quality, leading to missing values for “qualitative assessment” (*n* = 2), “number” (*n* = 6), and “angle” (*n* = 6).

In the final sample, “qualitative assessment” ranged from 0.25 to 100 on the VAS (*M* = 36.63, *SD* = 31.45), and the “number” of wrinkles varied from 0 to 5 wrinkles (*M* = 0.80, *SD* = 1.10). The “angle” ranged from 5.8° to 50.6° (*M* = 13.00, *SD* = 14.70).

For one outcome measure (“angle”) and two explanatory variables (“breed type”, “coat colour”), a subset of the full data set was used. For “angle” only pictures with at least one wrinkle (“number” ≥ 1) were included since an angle could only be measured if at least one wrinkle was identified, resulting in 427 pictures. For the analyses of “breed type” on the different outcome measures, all ponies (*n* = 7) and the horse without known breed were removed from the data set due to the small sample size, leading to a remaining sample of 916 pictures. For 902 pictures a “coat colour” could be assigned and these were therefore used for subsequent analyses.

### Intra- and Inter-observer Agreement

Results are given for all outcome measures including “brow raised” and “markedness” in case these outcome measures will be included in future studies. Comparison of first and second scoring of rater LS (intra-observer agreement) exceeded 0.9 for all continuous outcome measures (“qualitative assessment”: ICC _agreement_ = 0.90, with a 95% CI from 0.87 to 0.93; “brow raised”: ICC _agreement_ = 0.94 with a 95% CI from 0.92 to 0.96; “number”: ICC _agreement_ = 0.97, with a 95% CI from 0.96 to 0.98; “angle”: ICC _agreement_ = 0.97, with a 95% CI from 0.96 to 0.98) and 0.8 for the categorical outcome measure (“markedness”: κ = 0.92) with all *p*-values being highly significant (*p* < 0.001). Inter-observer agreement was slightly lower than within rater LS, but still exceeded 0.75 for all continuous outcome measures (“qualitative assessment”: ICC _agreement_ = 0.80, with a 95% CI from 0.70 to 0.87; “brow raised”: ICC _agreement_ = 0.84, with a 95% CI from 0.77 to 0.89; “number”: ICC _agreement_ = 0.78, with a 95% CI from 0.69 to 0.85; “angle”: ICC _agreement_ = 0.99, with a 95% CI from 0.98 to 0.99) and equalled 0.8 for the categorical outcome measure (“markedness”: κ = 0.80). Again all *p*-values were highly significant (*p* < 0.001).

### Assessment of the Outcome Measures

**Figure 2:**
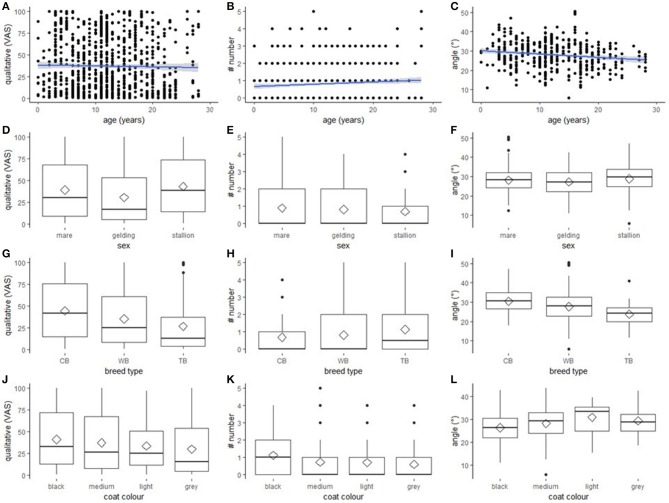
Effect of “age”, “sex”, “breed type”, and “coat colour” on the three outcome measures. The effect of “age”, “sex”, “breed type”, and “coat colour” on the outcome measures “qualitative assessment”, “number”, and “angle.” **(A–C)** scatter plots with regression line (method = lm, blue line) with 0.95 confidence intervals (grey). **(D–L)** boxplots with median (black line), mean (diamond), interquartile range (box), 1.5 × interquartile range (whiskers). **(A,D,G,J)** “qualitative assessment” assessed on Visual Analogue Scale. **(B,E,H,K)** “number” of wrinkles. **(C,F,I,L)** “angle” measured in degrees. **(G–I)** “breed type”: coldblood (CB), warmblood (WB), thoroughbred (TB). **(J-L)** “coat colour”: black or dark bay (dark), medium bay (medium), light bay and palomino (light), grey and white (grey).

All explanatory variables selected for inclusion in the respective final models are presented in Table 3. None of the selected variables had an effect on any of the outcome measures with one exception: “breed type” had a statistically significant effect on “angle” [*F*_(2, 114)_ = 8.25, *p* < 0.001; Figure 2]. *Post-hoc* tests revealed that thoroughbreds (*M* = 23.82, *SD* = 1.59) had a narrower “angle” than warmbloods (*M* = 28.00, *SD* = 0.60; *p* = 0.040) and coldbloods (*M* = 30.98, *SD* = 0.92; *p* < 0.001), and that warmbloods had a narrower “angle” than coldbloods (*p* = 0.022). Graphs for all outcome measures (“qualitative assessment”, “number”, “angle”) grouped by explanatory variables (“age”, “sex”, “breed type”, “coat colour”) can be found in Figure 2.

**Table 3:** Statistical models, explanatory variables to be included in the final model and results for the three outcome measures.

**Outcome measure**	**Statistical model**	**Selected explanatory variable(s)**	**Test statistic**	***p*-value**
Qualitative assessment	Linear mixed-effects model	Sex	*F*_(2, 161)_ = 2.35	0.097
		Breed type	*F*_(2, 161)_ = 1.31	0.343
		Coat colour	*F*_(3, 520)_ = 1.12	0.340
Number	Linear mixed-effects model	Sex	*F*_(2, 161)_ = 1.93	0.149
		Breed type	*F*(_2, 161)_ = 2.46	0.082
		Coat colour	*F*_(3, 516)_ = 0.23	0.903
Angle	Linear mixed-effects model	Breed type	*F*_(2, 114)_ = 8.25	0.001

### Side Effects

**Figure 3:**
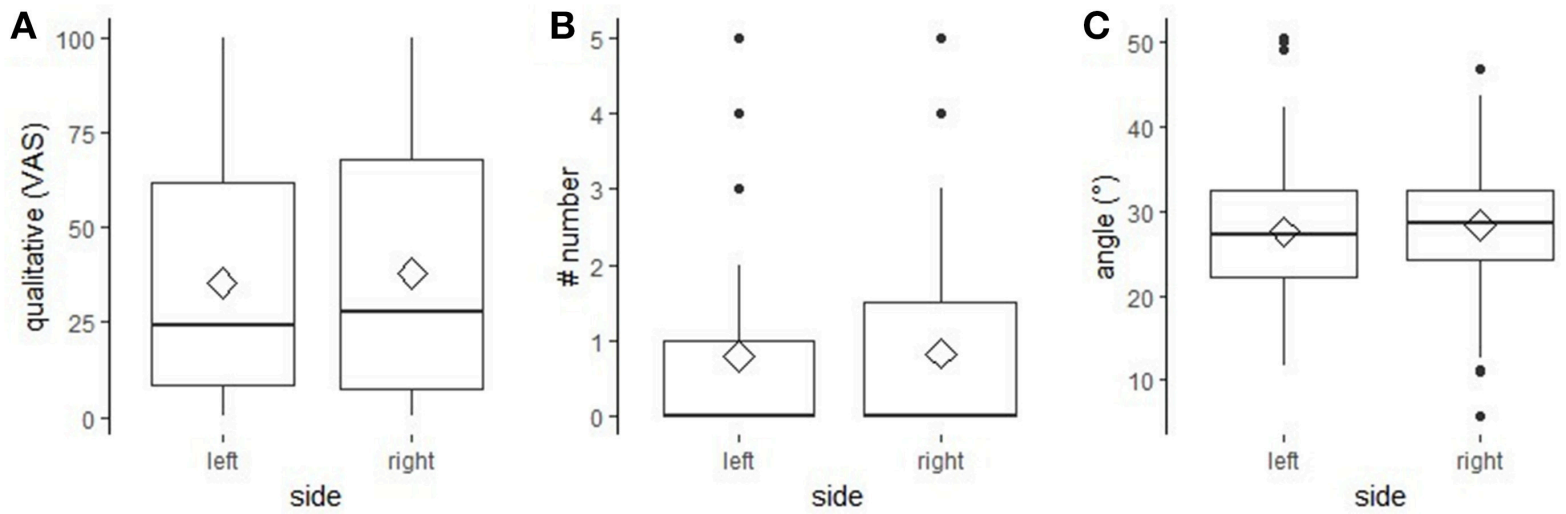
Effect of “side” on the three outcome measures. The effect of “side” (left, right) on the outcome measures “qualitative assessment” assessed on a Visual Analogue Scale **(A)**, “number” of wrinkles **(B)** and “angle” measured in degrees **(C)**. **(A–C)** boxplots with median (black line), mean (diamond), interquartile range (box), 1.5 × interquartile range (whiskers).

No statistically significant effect of “eye” on any of the three outcome measures was found [“qualitative assessment”: *F*_(1,179)_ = 3.06, *p* = 0.082; “number”: *F*_(1,353)_ = 0.41, *p* = 0.380; “angle”: *F*_(1,353)_ = 3.22, *p* = 0.370; Figure 3].

## Discussion

In the present study we investigated whether age, sex, breed type, body condition, and coat colour systematically affect eye wrinkle expression or its assessment in pictures taken of the left and the right hemiface of horses in a presumably neutral situation. Eye wrinkle expression was assessed using five outcome measures, all of which could be assessed highly reliable with respect to both intra- and inter-observer agreement. Some outcome measures were associated, therefore only “qualitative assessment”, “number”, and “angle” were further analysed. Similarly, “Body Condition Score” was strongly associated with the two explanatory variables “sex” and “breed type” and was thus not included in further analyses. “Breed type” influenced the width of the “angle”: thoroughbreds had a narrower “angle” than warmbloods and coldbloods, and warmbloods had a narrower “angle” than coldbloods. The three other explanatory variables (“age”, “sex”, and “coat colour”) did not affect any of the outcome measures, and eye wrinkle expression did not differ between the left and right eye.

### Characteristics of the Investigated Sample

We found substantial variability within each outcome measure, similar to what Hintze et al. (1) described for their presumably neutral control phases before the start of the experimental treatments. This variability may be explained by individual characteristics of horses independent of our tested characteristics, which did not account for the variation we found (with the exception of “breed type” accounting for differences in the “angle”). However, other explanations need to be considered as well. First, individual horses may have reacted differently to the halter, the human handling and/or the photographing. Even though we only took pictures when horses appeared to be calm and relaxed (standing still, head approximately at wither height), we cannot exclude that single individuals were slightly stressed by the procedure. Second, variation in eye wrinkle expression across horses could be caused by differences in underlying mood states, but this explanation is speculative since we did not assess mood in the present study.

Certain confounding effects (e.g., between “breed type” and “farm”, see below) could not be ruled out since our sample was not fully balanced across all explanatory variables. However, we counteracted this limitation as much as possible under non-experimental conditions by ensuring a relatively large sample for the different categories of “sex” (70 mares, 62 geldings, and 49 stallions), “breed type” (52 coldbloods, 104 warmbloods, and 17 thoroughbreds) and “age” (including young to very old horses).

### Relationship Between “Body Condition Score” and the Two Explanatory Variables “Breed Type” and “Sex”

Variation in “BCS” could be explained by the three “breed types”, with coldbloods having the highest scores, followed by warmbloods and thoroughbreds. This finding is consistent with what has been reported by Giles et al. (53), who found that breed was the risk factor most strongly associated with obesity in horses. In line with this, Visser et al. (54) found that coldbloods were more prone to develop a higher body condition score compared to thoroughbreds, which was also the case in our study. However, the association between “BCS” and “breed type” in our study could also be explained by confounds in our sample since most coldbloods were from Farm 7 and the management practices on a farm, especially the feeding regime, including the amount of feed and its nutritional value, can influence the body condition of horses. This confound may also explain the strong association between “sex” and “breed type” since most of the stallions on Farm 7 were coldbloods.

### Interpretation of the Results of the Outcome Measures

“Breed type” systemically affected the “angle” between the extensions of lines through both corners of the eye and through the topmost wrinkle. In our study, thoroughbreds had the narrowest “angle”, followed by warmbloods and coldbloods. “Angle” may be a measure of contraction of the levator anguli occouli and the corrugator supercilii muscles, with a wider angle reflecting a stronger contraction and a narrower angle reflecting a more relaxed muscle. To interpret “angle” as a measure of muscle contraction is plausible if differences are assessed, for example, when “angle” in a control situation is compared to “angle” in a treatment situation or when it is recorded continuously in video clips. In the present study, eye wrinkles were only assessed in a presumably neutral situation and in pictures, thus other explanations need to be discussed as well. Confounders may have affected our results with most coldbloods being derived from Farm 7. However, thoroughbreds came from six and warmbloods from all seven farms, which is why the confounding of “breed type” and “farm” cannot fully explain the variation in “angle.” Another confound that needs to be considered is the one between “breed type” and the different housing conditions of the horses. Whereas most thoroughbreds were kept either in groups, paddock boxes or boxes with daytime turnout, most of the coldbloods were housed in standard single horse boxes. Housing conditions may have affected mood and thus eye wrinkle expression with group-housed horses and horses with more space as well as physical contact to conspecifics having more relaxed muscles above the eye than horses kept in single boxes. However, our study was neither designed to study the effect of housing conditions on eye wrinkle expression, nor was mood investigated.

Beside the systematic effect of “breed type” on “angle”, there was no further effect of any of the explanatory variables on our outcome measures. This finding indicates that eye wrinkle expression can be assessed regardless of “age”, “sex”, and “coat colour”, while “breed type” should be considered in future studies. Our study does not give further insight into the relationship between emotion or mood and eye wrinkle expression, but it shows that eye wrinkle expression in horses cannot simply be explained by the investigated characteristics of the horses.

### Side Effects

No difference in eye wrinkle expression between the left and right eye area was found in a neutral situation, neither did Hintze et al. (1) find a difference in positively and negatively valenced situations. The results from both studies suggest that it is irrelevant which eye area is assessed; this could be advantageous if eye wrinkle expression is used in the future as an on-farm indicator of horses' emotional states.

## Conclusion

To our knowledge, this was the first study systematically investigating the effect of individual characteristics on eye wrinkle expression and its assessment in horses. We conclude that our eye wrinkle assessment scale can be used reliably and regardless of horses' age, sex, coat colour, and breed type (here with the exception of the “angle”). Thus, the adapted scale is a promising tool to assess eye wrinkles in horses, but to what extent these are systematically affected by mood or emotion or the interaction of mood and/or emotion with individual characteristics needs further investigation and validation.

## Data Availability

The datasets generated for this study are included in the Supplementary Material.

## Ethics Statement

The ethical guidelines of the International Society for Applied Ethology were respected while carrying out this experiment. For photographing, horses were loosely held on a halter (a normal routine for all horses used in this study) without any further manipulation. Horses from Farm 7 were additionally used in two larger studies, which were approved by the Cantonal Veterinary Office in Vaud, Switzerland (license numbers 2804 and 2804_1).

## Author Contributions

LS, KK, and SH conceived the study. LS and SH developed the methodology, collected the data, and wrote the manuscript. LS scored all pictures and performed the data analysis. All authors edited the manuscript, contributed to manuscript revision, read, and approved the submitted version.

### Conflict of Interest Statement

The authors declare that the research was conducted in the absence of any commercial or financial relationships that could be construed as a potential conflict of interest.
