# Supplementary material for: Sex and Age Don't Matter, but Breed Type Does—Factors Influencing Eye Wrinkle Expression in Horses
Source: Front Vet Sci. 2019 May 29;6:154. doi: 10.3389/fvets.2019.00154 (PMC6549476; doi:10.3389/fvets.2019.00154)
Supplement: Supplementary file 1 [file Table_1.DOCX]

Supplementary Material

# Supplementary Table

**Supplementary Table 1.** Information on the 181 horses included in this study. Breed type: CB = coldblood (n = 36), WB = warmblood (n = 106), TB = thoroughbred (n = 17), pony (n = 7), Breed: BW = Baden-Württemberg, Housing: box = single box (32.6 %), box w/ day pad = box with daytime paddock (38.1 %), Pbox w/ day pad = paddock box with daytime paddock (3.3 %), group = in group day and night (26.0 %).

| **Farm** | **Age (in years)** | **Sex** | **Breed type** | **Breed** | **Housing** |
| --- | --- | --- | --- | --- | --- |
| 1 | 11 | Gelding | WB | Knapstrubber | Box w/ day pad |
| 1 | 13 | Gelding | WB | Bavarian warmblood | Box w/ day pad |
| 1 | 6 | Gelding | TB | Paint x TB | Pbox w/ day pad |
| 1 | 28 | Mare | TB | Quarter horse x TB | Box w/ day pad |
| 1 | 17 | Mare | Pony | Lewitzer | Box w/ day pad |
| 1 | 19 | Mare | Pony | Pinto x pony | Pbox w/ day pad |
| 1 | 7 | Mare | WB | Quarter horse | Pbox w/ day pad |
| 2 | 8 | Gelding | Pony | German Riding Pony | Box w/ day pad |
| 2 | 22 | Gelding | Pony | German Riding Pony | Box w/ day pad |
| 2 | 7 | Gelding | TB | Quarter horse x TB | Box w/ day pad |
| 2 | 6 | Gelding | WB | Friesian | Box w/ day pad |
| 2 | 9 | Mare | WB | Friesian | Box w/ day pad |
| 3 | 23 | Gelding | TB | Galloper | Box w/ day pad |
| 3 | 8 | Gelding | WB | Bavarian warmblood | Box w/ day pad |
| 3 | 16 | Gelding | WB | BW warmblood | Box w/ day pad |
| 3 | 8 | Gelding | WB | German Sport Horse | Box w/ day pad |
| 3 | 13 | Gelding | WB | Hanoverian | Box w/ day pad |
| 3 | 15 | Gelding | WB | Hanoverian | Box w/ day pad |
| 3 | 16 | Gelding | WB | Hanoverian | Box w/ day pad |
| 3 | 20 | Gelding | WB | Oldenburger warmblood | Box w/ day pad |
| 3 | 12 | Gelding | WB | Saxony Anhaltiner WB | Box w/ day pad |
| 3 | 28 | Gelding | WB | Trakehner | Box w/ day pad |
| 3 | 27 | Gelding | WB | Trakehner x Holsteiner | Box w/ day pad |
| 3 | 11 | Mare | Pony | Haflinger | Box w/ day pad |
| 3 | 8 | Mare | WB | BW warmblood | Box w/ day pad |
| 3 | 24 | Mare | WB | BW warmblood | Box w/ day pad |
| 3 | 8 | Mare | WB | Friesian x WB | Box w/ day pad |
| 3 | 19 | Mare | WB | German Sport Horse | Box w/ day pad |
| 3 | 14 | Mare | WB | Oldenburger warmblood | Box w/ day pad |
| 3 | 22 | Mare | WB | Oldenburger warmblood | Box w/ day pad |
| 3 | 22 | Mare | WB | Oldenburger warmblood | Box w/ day pad |
| 3 | 8 | Mare | WB | Trakehner | Box w/ day pad |
| 3 | 21 | Mare | WB | Trakehner | Box w/ day pad |
| 4 | 12 | Gelding | TB | Arabian | Group |
| 4 | 21 | Gelding | TB | Galloper | Group |
| 4 | 6 | Gelding | TB | TB x Arabian | Group |
| 4 | 23 | Gelding | TB | TB x Arabian | Group |
| 4 | 5 | Gelding | WB | Andalusian | Group |
| 4 | 24 | Gelding | WB | German Sport Horse | Group |
| 4 | 22 | Mare | TB | Hispano Arabian | Group |
| 4 | 20 | Mare | WB | Polish WB x TB | Group |
| 4 | 13 | Mare | WB | Trakehner | Group |
| 5 | 4 | Gelding | WB | BW warmblood | Group |
| 5 | 7 | Gelding | WB | Oldenburger warmblood | Box w/ day pad |
| 5 | 7 | Gelding | WB | Hanoverian | Box w/ day pad |
| 5 | 6 | Gelding | WB | Andalusian | Box w/ day pad |
| 5 | 15 | Gelding | WB | Bavarian warmblood | Pbox w/ day pad |
| 5 | 10 | Gelding | WB | Friesian | Pbox w/ day pad |
| 5 | 9 | Gelding | WB | Hanoverian | Group |
| 5 | 24 | Gelding | WB | Hanoverian | Group |
| 5 | 7 | Mare | WB | Hanoverian | Pbox w/ day pad |
| 5 | 11 | Mare | WB | BW warmblood | Box w/ day pad |
| 6 | 11 | Gelding | WB | Lipizzaner | Box w/ day pad |
| 6 | 8 | Gelding | WB | Morgan | Box w/ day pad |
| 6 | 12 | Gelding | WB | Morgan | Box w/ day pad |
| 6 | 20 | Gelding | WB | Morgan | Box w/ day pad |
| 6 | 16 | Gelding | TB | Quarter horse x TB | Box w/ day pad |
| 6 | 9 | Gelding | WB | Quarter horse x WB | Box w/ day pad |
| 6 | 4 | Gelding | WB | Swedish warmblood | Box w/ day pad |
| 6 | 13 | Gelding | WB | Swedish warmblood | Box w/ day pad |
| 6 | 17 | Gelding | WB | Swedish warmblood | Box w/ day pad |
| 6 | 11 | Gelding | WB | Warmblood | Box w/ day pad |
| 6 | 16 | Gelding | WB | Friesian | Group |
| 6 | 1 | Gelding | WB | Lipizzaner | Group |
| 6 | 26 | Gelding | WB | PRE | Box w/ day pad |
| 6 | 2 | Gelding | WB | Warmblood | Group |
| 6 | 27 | Gelding | Pony | Shetland pony | Group |
| 6 | 1 | Gelding | WB | Lipizzaner | Group |
| 6 | 1 | Gelding | WB | Lipizzaner | Group |
| 6 | 7 | Mare | WB | Appaloosa | Box w/ day pad |
| 6 | 21 | Mare | WB | Hanoverian | Box w/ day pad |
| 6 | 4 | Mare | WB | Lipizzaner | Box w/ day pad |
| 6 | 14 | Mare | WB | Lipizzaner | Box w/ day pad |
| 6 | 17 | Mare | WB | Morgan | Box w/ day pad |
| 6 | 10 | Mare | WB | Paint horse | Box w/ day pad |
| 6 | 11 | Mare | WB | Quarter horse | Box w/ day pad |
| 6 | 11 | Mare | WB | Swedish warmblood | Box w/ day pad |
| 6 | 16 | Mare | WB | Warmblood | Box w/ day pad |
| 6 | 15 | Mare | CB | Draft x thoroughbred | Group |
| 6 | 20 | Mare | Pony | Connemara | Group |
| 6 | 2 | Mare | WB | Lipizzaner | Group |
| 6 | 11 | Mare | WB | Lipizzaner | Group |
| 6 | 11 | Mare | WB | Lipizzaner | Group |
| 6 | 15 | Mare | WB | Lipizzaner | Group |
| 6 | 17 | Mare | WB | Lipizzaner | Group |
| 6 | 6 | Mare | WB | Lipizzaner | Group |
| 6 | 10 | Mare | WB | Lusitano | Group |
| 6 | 18 | Mare | WB | Swedish warmblood | Group |
| 6 | 3 | Mare | WB | Warmblood | Group |
| 6 | 11 | Stallion | WB | Lipizzaner | Box w/ day pad |
| 6 | 15 | Stallion | WB | Lipizzaner | Box w/ day pad |
| 6 | <1 (4 months) | Stallion | WB | Lipizzaner | Group |
| 6 | <1 (5 months) | Stallion | WB | Lipizzaner | Group |
| 6 | 19 | Gelding | TB | Arabian | Box w/ day pad |
| 6 | 22 | Gelding | TB | Arabian | Box w/ day pad |
| 6 | 24 | Gelding | TB | Arabian | Box w/ day pad |
| 6 | 17 | Gelding | TB | Thoroughbred | Box w/ day pad |
| 6 | 20 | Gelding | WB | Appaloosa | Box w/ day pad |
| 6 | 18 | Gelding | WB | Dutch warmblood | Box w/ day pad |
| 6 | 18 | Gelding | WB | Dutch warmblood | Box w/ day pad |
| 6 | 12 | Gelding | WB | Friesian | Box w/ day pad |
| 6 | 10 | Gelding | WB | Lipizzaner | Box w/ day pad |
| 6 | 11 | Gelding | WB | Lipizzaner | Box w/ day pad |
| 6 | 8 | Gelding | WB | Morgan | Box w/ day pad |
| 6 | 12 | Gelding | WB | Morgan | Box w/ day pad |
| 6 | 20 | Gelding | WB | Morgan | Box w/ day pad |
| 6 | 16 | Gelding | WB | Quarter horse | Box w/ day pad |
| 6 | 9 | Gelding | WB | Quarter horse x WB | Box w/ day pad |
| 6 | 4 | Gelding | WB | Swedish warmblood | Box w/ day pad |
| 6 | 13 | Gelding | WB | Swedish warmblood | Box w/ day pad |
| 6 | 17 | Gelding | WB | Swedish warmblood | Box w/ day pad |
| 6 | 11 | Gelding | WB | Warmblood | Box w/ day pad |
| 6 | 16 | Gelding | WB | Friesian | Group |
| 6 | 1 | Gelding | WB | Lipizzaner | Group |
| 6 | 26 | Gelding | WB | PRE | Box w/ day pad |
| 6 | 2 | Gelding | WB | Warmblood | Group |
| 6 | 27 | Gelding | Pony | Shetland pony | Group |
| 6 | 1 | Gelding | WB | Lipizzaner | Group |
| 6 | 1 | Gelding | WB | Lipizzaner | Group |
| 6 | 7 | Mare | WB | Appaloosa | Box w/ day pad |
| 6 | 21 | Mare | WB | Hanoverian | Box w/ day pad |
| 6 | 4 | Mare | WB | Lipizzaner | Box w/ day pad |
| 7 | 3 | Gelding | CB | Franches-Montagnes | Box |
| 7 | 12 | Gelding | WB | Swiss warmblood | Box |
| 7 | 12 | Gelding | WB | Swiss warmblood | Box |
| 7 | 16 | Gelding | WB | Hanoverian | Box |
| 7 | 3 | Mare | CB | Franches-Montagnes | Box |
| 7 | 3 | Mare | CB | Franches-Montagnes | Box |
| 7 | 3 | Mare | WB | Swiss warmblood | Box |
| 7 | 3 | Mare | CB | Franches-Montagnes | Box |
| 7 | 17 | Mare | WB | Swiss warmblood | Group |
| 7 | 12 | Mare | WB | Swiss warmblood | Box w/ day pad |
| 7 | 6 | Mare | WB | Swiss warmblood | Box w/ day pad |
| 7 | 12 | Mare | WB | Swiss warmblood | Group |
| 7 | 3 | Mare | CB | Franches-Montagnes | Box |
| 7 | 3 | Mare | CB | Franches-Montagnes | Box |
| 7 | 15 | Mare | WB | Swiss warmblood | Group |
| 7 | 4 | Mare | WB | Swiss warmblood | Group |
| 7 | 13 | Mare | WB | Swiss warmblood | Group |
| 7 | 9 | Mare | WB | Swiss warmblood | Group |
| 7 | 15 | Mare | WB | Swiss warmblood | Group |
| 7 | 11 | Mare | CB | Franches-Montagnes | Group |
| 7 | 3 | Mare | CB | Franches-Montagnes | Box |
| 7 | 12 | Mare | CB | Franches-Montagnes | Group |
| 7 | 13 | Mare | WB | Swiss warmblood | Box w/ day pad |
| 7 | 3 | Mare | CB | Franches-Montagnes | Box |
| 7 | 12 | Mare | WB | Swiss warmblood | Group |
| 7 | 19 | Mare | WB | Swiss warmblood | Group |
| 7 | 14 | Mare | CB | Franches-Montagnes | Group |
| 7 | 13 | Mare |  |  | Box w/ day pad |
| 7 | 12 | Mare | WB | Swiss warmblood | Box |
| 7 | 11 | Mare | TB | French Trotter | Group |
| 7 | 11 | Mare | TB | French Trotter | Group |
| 7 | 13 | Mare | CB | Franches-Montagnes | Group |
| 7 | 6 | Mare | WB | Selle français | Box w/ day pad |
| 7 | 5 | Mare | WB | Selle français | Box |
| 7 | 23 | Stallion | WB | Holsteiner | Box |
| 7 | 17 | Stallion | CB | Franches-Montagnes | Box |
| 7 | 12 | Stallion | WB | Hanoverian | Box |
| 7 | 10 | Stallion | CB | Franches-Montagnes | Box |
| 7 | 6 | Stallion | WB | Hanoverian | Box |
| 7 | 20 | Stallion | CB | Franches-Montagnes | Box |
| 7 | 4 | Stallion | WB | Belgian WB | Box |
| 7 | 10 | Stallion | CB | Franches-Montagnes | Box |
| 7 | 9 | Stallion | CB | Franches-Montagnes | Box |
| 7 | 11 | Stallion | CB | Franches-Montagnes | Box |
| 7 | 20 | Stallion | WB | Selle français | Box |
| 7 | 8 | Stallion | CB | Franches-Montagnes | Box |
| 7 | 6 | Stallion | CB | Franches-Montagnes | Box |
| 7 | 12 | Stallion | CB | Franches-Montagnes | Box |
| 7 | 11 | Stallion | CB | Franches-Montagnes | Box |
| 7 | 12 | Stallion | CB | Franches-Montagnes | Box |
| 7 | 2 | Stallion | CB | Franches-Montagnes | Box |
| 7 | 12 | Stallion | CB | Franches-Montagnes | Box |
| 7 | 14 | Stallion | CB | Franches-Montagnes | Box |
| 7 | 5 | Stallion | CB | Franches-Montagnes | Box |
| 7 | 5 | Stallion | CB | Franches-Montagnes | Box |
| 7 | 11 | Stallion | CB | Franches-Montagnes | Box |
| 7 | 7 | Stallion | CB | Franches-Montagnes | Box |
| 7 | 6 | Stallion | CB | Franches-Montagnes | Box |
| 7 | 4 | Stallion | CB | Franches-Montagnes | Box |
| 7 | 5 | Stallion | CB | Franches-Montagnes | Box |
| 7 | 6 | Stallion | CB | Franches-Montagnes | Box |
| 7 | 22 | Stallion | CB | Franches-Montagnes | Box |
| 7 | 13 | Stallion | CB | Franches-Montagnes | Box |
| 7 | 8 | Stallion | CB | Franches-Montagnes | Box |
| 7 | 11 | Stallion | CB | Franches-Montagnes | Box |
| 7 | 12 | Stallion | CB | Franches-Montagnes | Box |
